# UL16-Binding Protein 1 Induced HTR-8/SVneo Autophagy via NF-*κ*B Suppression Mediated by TNF-*α* Secreted through uNK Cells

**DOI:** 10.1155/2020/9280372

**Published:** 2020-06-13

**Authors:** Jing Liu, Guang Song, Tao Meng, Ge Zhao

**Affiliations:** ^1^Department of Obstetrics, The First Affiliated Hospital of China Medical University, Shenyang, China; ^2^Department of Ultrasound, Shengjing Hospital of China Medical University, Shenyang, China

## Abstract

UL16-binding protein 1(ULBP1) has been reported to inhibit trophoblast invasion through the modification of secretion functions of uNK cells in the previous study, but its mechanisms remain unclear. In this study, we investigated the related mechanism by which upregulated ULBP1 expression impaired trophoblast invasion. We found that conditioned media with ULBP1 increased autophagy in HTR-8/SVneo, and anti-TNF-*α*-neutralizing antibody rescued the autophagy caused by the conditioned medium. We further found TNF-*α* induced autophagy in trophoblast cells in a dose-dependent way and accompanied by a decreased activity of nuclear factor-kappa B (NF-*κ*B). Inhibition of NF-*κ*B activation by chemical inhibitor augmented these autophagic responses to TNF-*α* in the cells. In addition, interruption NF-*κ*B caused a significant decrease in HTR-8/SVneo invasion and enhanced the inhibition effect of TNF-*α* on HTR-8/SVneo invasion. Taken together, these findings suggest that TNF-*α* is able to regulate autophagic activity via suppressing NF-*κ*B, which might be the mechanism related to ULBP1 in preeclampsia pathogenesis.

## 1. Introduction

Preeclampsia (PE) is a condition that begins during pregnancy and is the leading cause of maternal and fetal morbidity and mortality. To date, the etiology and pathogenic mechanisms of PE remain unclear. Autophagy is a process of self-degradation of cellular components; double-membrane autophagosomes sequester organelles and fuse with lysosomes so that the contents can be digested by lysosomal enzymes [1]. The functions of autophagy have been seen as an adaptive response to survival under oxidative stress and other environmental stressors [2]. Recent studies have indicated that overactivation of autophagy could promote cellular dysfunction through excessive degradation of essential cellular constituents [3]. Moreover, autophagy has been implicated in the pathogenesis of PE [4]. Under physiological conditions of low oxygen, autophagy is essential for extravillous trophoblast (EVT) invasion and vascular remodeling [5]. But the increased autophagy activity in trophoblasts was found to affect trophoblast invasion and placental vasculature, which suggests that excessive autophagy is an important trait in the placenta during PE pathogenesis [6]. Therefore, investigations on the molecular mechanisms related to trophoblastic autophagy activity will help to understand the pathogenesis of PE.

In the previous studies, we have demonstrated that ULBP1 was upregulated in PE placentas and it inhibited trophoblast invasion through the modification of secretion functions of uterine natural killer (uNK) cells, but the specific mechanism has not been investigated [7]. Tumor necrosis factor-alpha (TNF-*α*) is one of the cytokines secreted by uNK, and it was elevated after ULBP1 binding to NKG2D on uNK cells [7]. As a pleiotropic cytokine, TNF-*α* mediates a broad range of proinflammatory activities, cell proliferation, differentiation, and death [8, 9] and it also involves autophagy. Oh et al. found that TNF-*α* increased the expression of LC3-II in the trophoblast cell [10]. Nevertheless, which mechanisms implicated in it and whether the autophagy induced by TNF-*α* is related to trophoblast cell invasion remain unclear.

NF-*κ*B is one of the most important pathways which can be initiated after stimulation with TNF-*α* [11]. As a transcriptional factor, NF-*κ*B is activated by multiple conditions including hypoxia, inflammatory cytokines, and pathogen infections [12]. The activation of the NF-*κ*B pathway leads to the release of p65/p50 subunits from the NF-*κ*B/I*κ*B complex and then the translocation of p65/p50 heterodimers to the nucleus to execute NF-*κ*B function [13]. NF-*κ*B has been reported to play a crucial role in autophagy in the development of human cancers [14, 15]. To our current knowledge, no studies have addressed the effect of NF-*κ*B on trophoblastic autophagy and invasion.

Considering the relationship between the NF-*κ*B and TNF-*α* signaling pathways and the role of NF-*κ*B in autophagy, we hypothesized that the inhibition effect of ULBP1 on trophoblast cell invasion is related to autophagy activation, which was mediated by NF-*κ*B via TNF-*α* secreted through uNK cells. In this study, we aimed to investigate our hypothesis. Combined with our previous research, the present results demonstrate a new role of ULBP1 playing in PE and a novel molecular pathway leading to compromised trophoblast invasion. This study may help to better understand the pathogenesis of PE.

## 2. Methods and Methods

### 2.1. Sample Preparation

Ethical approval was granted by the Ethics Committee of The First Affiliated Hospital of China Medical University (Shenyang, China), and methods were carried out in accordance with the committee guidelines. Informed consent was obtained from all participating patients. Decidual samples were obtained from women undergoing elective surgical termination of pregnancy at 12–14 weeks of gestation (as determined by ultrasound measurement of crown-rump length or biparietal diameter). Following collection, the decidual tissue was immediately suspended in sterile saline, transported to the laboratory, and washed two to three times in sterile phosphate-buffered saline (PBS) to remove excess blood.

### 2.2. uNK Cell Isolation

Total decidual cell isolates and purified CD56+ CD3- uNK cell isolates were prepared by enzymatic disaggregation and immunomagnetic selection (MACS) as previously described [16, 17]. Briefly, the decidual tissue was finely minced, incubated in DNase/collagenase, allowed to adhere overnight, and either used as total decidual cell suspensions or subjected to positive immunomagnetic selection (MidiMACS; Miltenyi Biotec GmbH, Bergisch Gladbach, Germany) with an NK Cell Isolation kit (Miltenyi Biotec GmbH) to obtain uNK cell suspensions. UNK cells were plated on a 24-well plate at 5 × 10^4^ cells/well in 600 *μ*l RPMI-1640 medium supplemented with 1,000 U/ml penicillin, 1 mg/ml streptomycin, 2 mM L-glutamine, and 10% fetal bovine serum (HyClone; GE Healthcare Life Sciences, USA), with or without 5 *μ*g/ml recombinant ULBP1-Fc chimera protein (Sino Biological Inc., Beijing, China), and incubated for 72 h in a standard 37°C environment (5% CO_2_). Cell viability was routinely tested by Trypan blue exclusion. Viability, which was calculated as viability = (total cells − dead cells)/total cells, was 80%–90% after 72 h of cell culture. The cell-free conditioned medium was removed and stored at -20°C.

### 2.3. Cell Culture and Treatment

EVT cell line HTR-8/SVneo cells were purchased from the American Type Culture Collection (ATCC) and cultured in RPMI-1640 medium (HyClone; GE Healthcare Life Sciences, Logan, UT, USA) supplemented with 10% FBS in a standard 37°C environment (5% CO2) in an air incubator. The different uNK cell conditioned media (cultured with or without the ULBP1 protein, 33% *v*/*v*) was added to GFP-LC3-labeled HTR-8/SVneo cell culture media with or without 5 *μ*g/ml anti-TNF-*α* (Cat. no. MAB610, R&D Systems, Minneapolis, MN, USA) to investigate the autophagy.

### 2.4. Measurement of Autophagy

HTR-8/SVneo cells were infected with pSELECT-GFP-LC3 adenovirus (MOI = 100) (GM-1314R203H, Genomeditech, Shanghai, China). 24 h after transfection, the cells were cultured in different conditions for another 24 h. Then, the fluorescence of GFP-LC3 was observed under a fluorescence microscope (OLYMPUS, IX53). Cells with five or more intense GFP-LC3 puncta were considered autophagic, whereas those with diffuse cytoplasmic GFP-LC3 staining were considered nonautophagic. The percentages of GFP-LC3-positive cells were counted in at least 100 cells.

### 2.5. Blockade of NF-*κ*B Signaling

GFP-LC3-labeled HTR-8/SVneo cells were plated at a density of 1 × 10^6^ cells/well in six-well tissue culture plates cultured for 24 h with different doses of TNF-*α* (300-01A, PeproTech, Rocky Hill, NJ, USA) (final concentration: 0 ng/ml, 10 ng/ml, and 20 ng/ml). The NF-*κ*B-specific inhibitor pyrrolidine dithiocarbamate (PDTC) (10 *μ*M) (Sigma, St.Louis, MO, USA) was given to the HTR-8/SVneo 1 h prior to the administration of TNF-*α*.

### 2.6. Western Blotting

HTR-8/SVneo cells were lysed in a tissue protein extraction reagent (Nanjing KeyGen Biotech Co., Ltd., Nanjing, China), and protein concentrations were determined using an Enhanced BCA Protein Assay kit (Beyotime Institute of Biotechnology, Beijing, China). A total of 20 *μ*g of proteins was separated by 10% SDS-PAGE and transferred onto a polyvinylidene difluoride membrane (Merck Millipore, Darmstadt, Germany). Membranes were blocked for 1 h at room temperature with TBST (50 mM Tris-HCl, 150 mM NaCl, 0.05% Tween-20, and pH 7.0) containing 5% nonfat dry milk and incubated overnight at 4°C with the following primary antibodies: anti-phosphorylated-NF-*κ*B p65 (p-NF-*κ*B p65) (1 : 500, sc-136548, Santa Cruz Biotechnology Inc., Dallas,TX, USA), anti-LC3B antibody (1 : 1000, L7543, Sigma, St. Louis, MO, USA), anti- p62/SQSTM1 (1 : 1000, P0067, Sigma, St. Louis, MO, USA), and anti-*β*-actin antibody (1 : 8000, ab8226, Abcam, USA). The membranes were washed with TBST three times for 10 min, followed by incubation with horseradish-peroxidase-labeled secondary antibodies (1 : 5000, WL01845, Wanleibio, Shenyang, China) for 1 h at room temperature. Protein expression was visualized using an enhanced chemiluminescence system (Tanon5200; Tanon Science and Technology Co., Ltd., Shanghai, China). Bands were analyzed using densitometry with ImageJ software (version 1.46r; National Institutes of Health, Bethesda, MA, USA).

### 2.7. Transmission Electron Microscopy

After culturing with different conditions, HTR-8/SVneo cells were fixed with PBS containing 3% glutaraldehyde and then postfixed with PBS containing 1% OsO4. The samples were dehydrated in graded alcohol, embedded in Durcupan resin, and sectioned. Ultrathin sections were stained with uranyl acetate and lead citrate and examined by an HT7700 transmission electron microscope (Hitachi, Japan).

### 2.8. Invasion Assay

A total of 1 × 10^5^ HTR-8/SVneo cells, with or without pretreatment with PDTC, in 200 *μ*l of serum-free medium were placed in the upper chamber of an 8 *μ*m Transwell plate (Costar, New York, NY, USA). Inserts were precoated with 80 *μ*l Matrigel matrix (1 : 9, BD Biosciences, San Jose, CA, USA). The lower chamber contained 600 *μ*l of complete culture medium (RPMI-1640 containing 10% FBS) with or without 10 ng/ml TNF-*α*. Cells in the chamber were cultured for 24 h, and the cells on the filter were methanol fixed for 15 min. Finally, the number of cells was counted by capturing images of the membrane with an Olympus CKX41 microscope (Olympus Corp.). Migrated cells were counted in 10 random fields using ImageJ software.

### 2.9. Statistical Analysis

All the presented data and results were confirmed in at least three independent experiments. The data are expressed as the mean ± S.D. The data were analyzed by one-way ANOVA using Statistics Package for Social Science (SPSS) software (version 13.0; SPSS, Chicago, IL, USA), and the statistical comparisons were made by the least significant difference (LSD) post hoc test. *P* < 0.05 was considered statistically significant.

## 3. Results

### 3.1. The Increased Autophagy in HTR-8/SVneo Cocultured with Conditioned Media with ULBP1 Is Mediated by TNF-*α*

**Figure 1:**
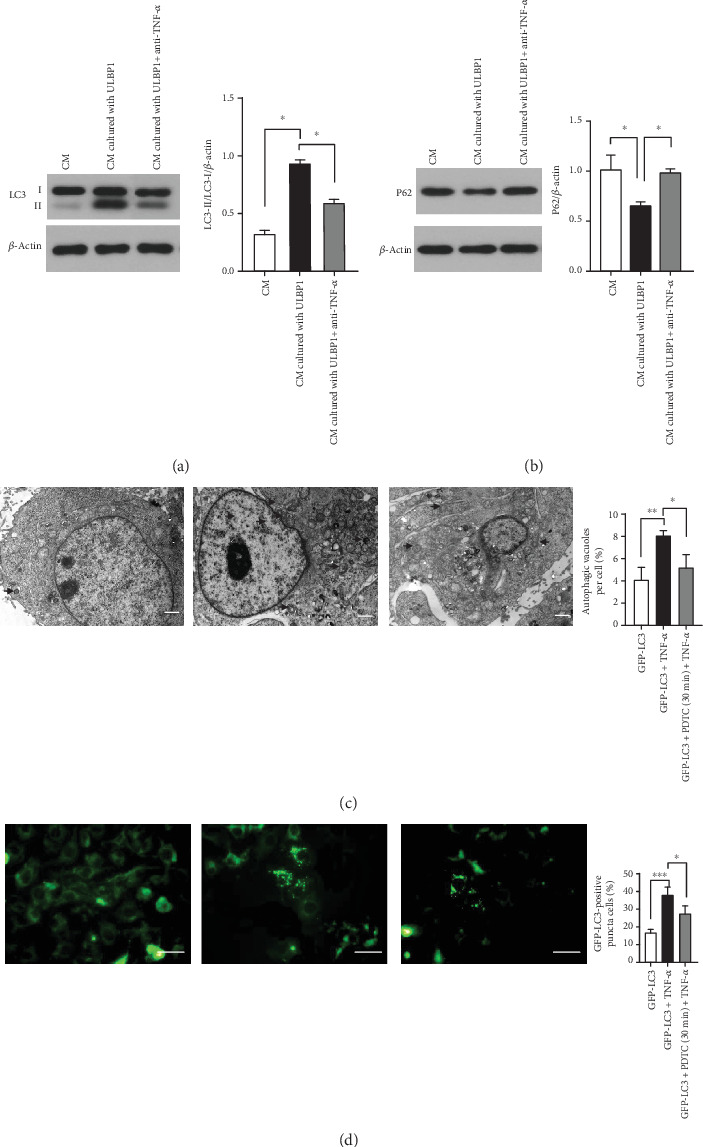
The increased autophagy in HTR-8/SVneo cocultured with conditioned media with ULBP1 is mediated by TNF-*α*. (a, b) The expression of LC3-II/LC3-I was significantly increased and P62 was significantly decreased in the conditioned media with the ULBP1 group (every two groups have significant difference). Following the addition of the anti-TNF-*α* neutralizing antibody to uNK cell culture supernatants with ULBP1, levels of LC3-II/I were significantly decreased and P62 was significantly increased compared to the conditioned media with the ULBP1 group (every two groups have significant difference). (c) Representative images of HTR-8/SVneo cells in different condition media observed by TEM. Arrows indicate the autophagosome. (d) Autophagic activities were observed using a fluorescence microscope. (^∗^*P* < 0.05, ^∗∗^*P* < 0.01, and ^∗∗∗^*P* < 0.001).

After cocultured with conditioned media with ULBP1, we evaluated the protein levels of LC3-II/I and P62 by Western blotting in HTR-8/SVneo. As shown in Figures 1(a) and 1(b), the expression of LC3-II/I was significantly increased and P62 was significantly decreased in the conditioned media with the ULBP1 group (*P* < 0.05). Following the addition of the anti-TNF-*α*-neutralizing antibody to conditioned media cultured with ULBP1, levels of LC3-II/I were significantly decreased and P62 was significantly increased compared to the conditioned media cultured with the ULBP1 group in the absence of the anti-TNF-*α*-neutralizing antibody (*P* < 0.05). The number of autophagosomes in HTR-8/SVneo treated with conditioned media cultured with ULBP1 in the presence or absence of anti-TNF-*α*-neutralizing antibody was shown in Figure 1(c). The distribution of LC3 was observed as shown in Figure 1(d).

### 3.2. TNF-*α* Induced HTR-8/SVneo Cell Autophagy

**Figure 2:**
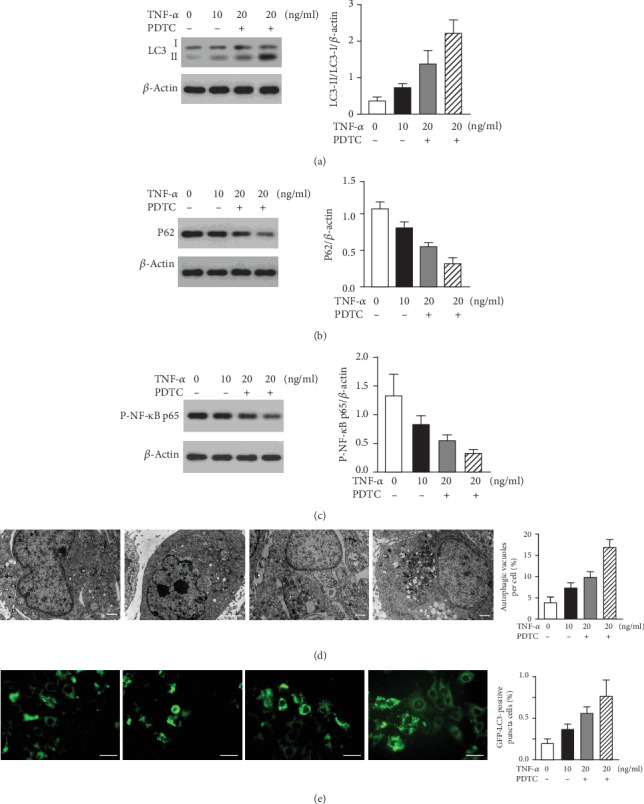
TNF-*α*-induced HTR-8/SVneo cell autophagy in a dose-dependent way via suppressing NF-*κ*B activity. (a, b) The expression of LC3-II/I was significantly increased and P62 was significantly decreased within a high dose of TNF-*α* (every two groups have significant difference). (c) NF-*κ*B inhibitor PDTC significantly increased the TNF-*α*-induced autophagy (every two groups have significant differences). (d) Representative images of HTR-8/SVneo cells in different condition media observed by TEM. Arrows indicate the autophagosome. (e) Autophagic activities were observed using a fluorescence microscope.

To further confirm the role of TNF-*α* in autophagy in HTR-8/SVneo, we administered TNF-*α* in different doses. Compared with 10 ng/ml TNF-*α* treatment, the expression of LC3-II/I was significantly increased and P62 was significantly decreased with 20 ng/ml administration of TNF-*α* (Figures 2(a) and 2(b)). The number of autophagosomes in HTR-8/SVneo treated with different doses of TNF-*α* was shown in Figure 2(d). The distribution of LC3 was observed as shown in Figure 2(e).

### 3.3. NF-*κ*B Activity Was Involved in TNF*α*-Induced HTR-8/SVneo Cell Autophagy

To investigate the mechanisms by which TNF-*α* promotes HTR-8/SVneo cell autophagy, we assessed the activity of NF-*κ*B. The expression of p-NF-*κ*B p65 was decreased with TNF-*α* administration (Figure 2(c)). We also found that inhibition of NF-*κ*B activation by using the NF-*κ*B inhibitor PDTC significantly increased the TNF-*α*-induced autophagy. The number of autophagosomes in HTR-8/SVneo under different culture conditions was shown in Figure 2(d). The distribution of LC3 was observed as shown in Figure 2(e).

### 3.4. Suppression of NF-*κ*B Promoted TNF-*α*-Induced HTR-8/SVneo Invasion Impairment

**Figure 3:**
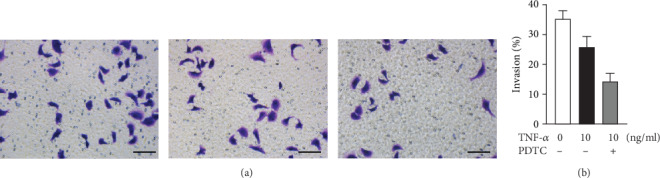
Suppression of NF-*κ*B promoted TNF-*α*-induced HTR-8/SVneo invasion impairment. (a) Representative images of invaded cells with or without pretreatment with PDTC cultured with different concentrations of TNF-*α* in the Transwell invasion assay. (b) Statistical bar graphs exhibited that the suppression of NF-*κ*B promoted TNF-*α*-induced HTR-8/SVneo invasion impairment in a Transwell invasion assay.

We examined the role of NF-*κ*B in TNF-*α*-induced HTR-8/SVneo invasion impairment. TNF-*α* inhibited the HTR-8/SVneo invasion. Compared with the TNF-*α* alone treatment group, interruption NF-*κ*B caused a significant decrease in HTR-8/SVneo invasion and enhanced the inhibition effect of TNF-*α* on HTR-8/SVneo invasion (Figure 3).

## 4. Discussion

In this study, we observed that uNK cell conditioned media cultured with ULBP1 significantly increased the protein ratio of LC3-II/LC3-I and decreased the expression of P62 in HTR-8/SVneo. Our fluorescence microscope and TEM images also demonstrated the fluorescence intensity of LC3, and the number of autophagosomes is increased in HTR-8/SVneo cocultured with conditioned media with ULBP1. We suggested that conditioned media cultured with ULBP1 enhanced autophagy in HTR-8/SVneo, and the effect was mediated by TNF-*α*. Our study further demonstrated that TNF-*α*-induced HTR-8/SVneo cell autophagy in a dose-dependent way and established that NF-*κ*B played a protective role in TNF-*α*-induced autophagy.

Autophagy is a biological process that cell degrades cellular components through the lysosomal pathway for survival in response to starvation. The induction of autophagy provides the cell with molecular building blocks and energy. However, excessive autophagy or autophagic dysfunction can induce serious illnesses. Autophagy is thought to be involved in early human placentation [18]. A number of studies have reported that typical markers of autophagy, LC3, and Beclin-1 expression, are significantly increased in placentas from pregnancies complicated by PE and intrauterine growth restriction (IUGR), suggesting that autophagy plays a key role in the pathogenesis of these diseases [5, 18]. Our previous study already showed that uNK cell conditioned media cultured with ULBP1 inhibited trophoblast cell invasion [7]. ULBP1 could downregulate the expression of NKG2D on uNK cells and then increase the secretion of cytokines of uNK cells. It was indicated that ULBP1 disturbs the reproductive balance on the maternal-fetal surface, contributing to the pathogenesis of PE. The represent study is aimed at investigating further the mechanism by which ULBP1 contributes to PE pathogenesis. We demonstrated that the uNK cell conditioned media cultured with ULBP1 increased autophagy in trophoblast cells in the current study.

The secreted function of uNK cells plays an important role in a successful pregnancy [19, 20]. As an active ligand of NK cells, ULBP1 leads to activate the secreted function of uNK cells. The imbalance on maternal-fetal interface results in suppressing EVT cell invasion, which is the feature of PE. The disturbance of secreted function of uNK cells could also be associated with the increased autophagy in HTR-8/SVneo cells.

Autophagy has attracted extensive attention in recent years due to the effects on various physiological and pathological processes. It is an evolutionarily conserved self-digestive process that targets intracellular substrates for lysosomal degradation and recycling in response to stress and other environmental signals [21–23], including nutrient deprivation, hypoxia, or infection. Many cytokines are also involved in controlling autophagy, such as IL-2 and TNF-*α*, and are generally considered autophagy inducers [24].

In this study, we found that the anti-TNF-*α*-neutralizing antibody significantly rescued the autophagy caused by a conditioned medium and further demonstrated that TNF-*α* could induce trophoblast cell autophagy in a dose-dependent way. TNF-*α* is a multifunctional cytokine, and it has various roles in the organism. It can induce more than five pathways and take part in inflammation, apoptosis, proliferation, or morphogenesis, In addition, studies reported that TNF-*α* is a critical regulator of autophagy in many diseases [25, 26]. The role of TNF-*α* in trophoblast cell autophagy in our study was consistent with the previous study [10]. However, the mechanism of TNF-*α*-induced autophagy in trophoblast cells requires to be investigated.

NF-*κ*B plays a crucial role in inflammatory and immune responses, as well as cell proliferation and survival [27, 28]. As a ubiquitous transcription factor, NF-*κ*B has been implicated in the control of apoptosis and autophagy [29, 30]. The NF-*κ*B family of inducible transcription factors is activated in response to a variety of stimuli. Amongst the best characterized inducers of NF-*κ*B are members of the TNF family of cytokines. Typically, in most unstimulated cells, NF-*κ*B is sequestered in the cytoplasm by binding to the inhibitor of NF-*κ*B (I*κ*B). The activation of NF-*κ*B has been reported to involve in autophagy in several diseases [31, 32]. Djavaheri-Mergny et al. found that the activation of NF-*κ*B mediates the repression of autophagy, which is a cell death mechanism in TNF-*α*-treated Ewing sarcoma cells [31]. In this study, we showed that inhibition of NF-*κ*B with PDTC increased TNF-*α*-induced autophagy in the trophoblastic cell, suggesting that NF-*κ*B had a protective role in TNF-*α*-induced autophagy. This was also supported by the work of Ye et al. [33]. We further demonstrated that PDTC augmented the inhibition effect of TNF-*α*-induced trophoblastic cell invasion. It is apparent that NF-*κ*B is a positive regulator in trophoblast invasion impairment induced by TNF-*α*. As pointed out above, the autophagy induced by TNF-*α* via suppressing NF-*κ*B is associated with the trophoblast invasion.

## 5. Conclusions

In conclusion, we investigated the mechanism related to ULBP1 in PE pathogenesis, suggesting the important role of TNF-*α* stimulated by ULBP1. To the best of our knowledge, the current study is the first to show that TNF-*α* induced autophagy via the suppression of NF-*κ*B, which resulted in trophoblast invasion impairment. These findings provide new evidence for further understanding the molecular mechanisms between PE and autophagy.

## Data Availability

The data used to support the findings of this study are included in the article.
